# A small window into the status of malaria in North Korea: estimation of imported malaria incidence among visitors from South Korea

**DOI:** 10.4178/epih.e2020068

**Published:** 2020-11-21

**Authors:** Jisun Sung, Hae-Kwan Cheong, Ah-Young Lim, Jong-Hun Kim

**Affiliations:** Department of Social and Preventive Medicine, Sungkyunkwan University School of Medicine, Suwon, Korea

**Keywords:** Malaria, *Plasmodium vivax*, Transboundary transmission, Surveillance, Democratic People’s Republic of Korea, Republic of Korea

## Abstract

**OBJECTIVES:**

This study aimed to develop hypotheses on trends in malaria incidence in North Korea using malaria incidence among South Korean visitors to North Korea.

**METHODS:**

The number of South Korean tourists who visited Mount Kumgang from 2000 to 2008 and the number of South Korean employees at the Kaesong Industrial Complex from 2005 to 2015 were obtained from the Korean Statistical Information Service. The number of malaria cases among South Koreans who visited North Korea was obtained from a previous report. The incidence of malaria per 100,000 person-years was calculated using these data and compared with the malaria incidence in North Korea derived from published articles.

**RESULTS:**

A high incidence of malaria in 2001 and a sharp decline in the following years were observed in both South and North Korean data. Since then, North Korean data showed a relatively low and stable incidence, but the incidence among South Koreans visiting North Korea increased in 2006. Considering the trends in mass primaquine preventive treatment, floods, and economic growth rate, the incidence of malaria may have increased in North Korea in 2006. Since 2009, the incidence of malaria decreased gradually according to both South and North Korean data.

**CONCLUSIONS:**

The trends of malaria incidence in North Korea could be reflected through its incidence among South Koreans who visited North Korea. For future inter-Korean collaboration aiming to eradicate malaria, we propose that a North Korean malaria monitoring system be established applying this method.

## INTRODUCTION

Malaria, which was eradicated in the Korean peninsula in the 1970s, re-emerged in South Korea and North Korea in the 1990s [1]. In South Korea (the Republic of Korea), the first case was reported in soldiers adjacent to the Demilitarized Zone (DMZ) in northern Gyeonggi Province in 1993. The number of cases increased, peaked at 4,142 in 2000, and then gradually decreased and recently stabilized at approximately 500-600 annually [2]. In North Korea (the Democratic People’s Republic of Korea), the number of cases increased explosively after 2,100 cases were reported in 1998, peaked at about 300,000 in 2001, and steadily decreased; 3,598 cases were reported in 2018 [3,4]. The re-emergence of *Plasmodium vivax* malaria in both South Korea and North Korea has mainly affected the areas bordered by the DMZ, that is, Incheon, Gyeonggi Province, and Gangwon Province in South Korea and Kaesong, South Hwanghae Province, North Hwanghae Province, and Kangwon Province in North Korea. As the temporal and spatial trends of malaria in South Korea and North Korea were closely related, monitoring the incidence of malaria in North Korea is important for planning malaria elimination in South Korea [5].

According to the World Health Organization (WHO) country cooperation strategy report [6], North Korea’s infectious disease surveillance and outbreak response is carried out through a network of hygiene and anti-epidemic stations that have been established at the central/province/county level under the Ministry of Public Health. Household doctors in charge of the designated area are responsible for diagnosis and reporting at the community level. Due to the nature of the socialist system, the reporting system may be considered to be robust. However, it is questionable whether the surveillance system works properly because of continuing shortages of medical resources [7]. In a study that assessed the national surveillance system for malaria in the Asia-Pacific region, Mercado et al. [8] pointed out that malaria incidence data in Asia-Pacific countries were likely to be missing information and that the WHO annual World Malaria Report may have uncertainty. According to a previous study, which evaluated the quality of malaria data in North Korea, most malaria cases reported to the WHO in the early 2000s were clinically diagnosed; only about 50% were confirmed by microscopic examination [5]. In terms of malaria incidence prior to 2011, the number reported to the WHO by the government of North Korea differed by more than 1,000 cases from the total number of cases of all provinces according to the aforementioned study. The quality of North Korean reported data is thought to have improved since 2011, as the diagnostic accuracy was enhanced through the supply of microscopes in the 2000s and stable financial support from the Global Fund from 2010.

**Figure 1.**
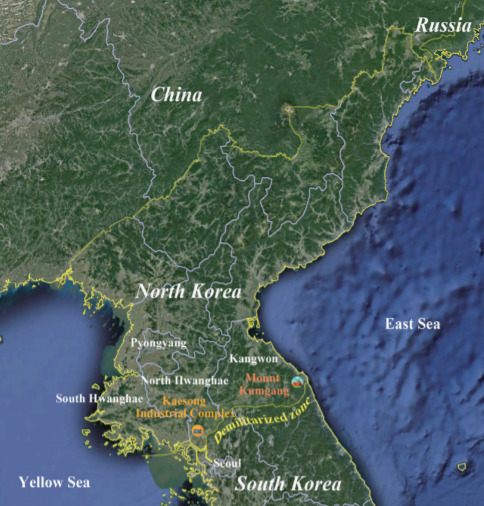
Location of Kaesong Industrial Complex and Mount Kumgang.

When it is difficult to obtain accurate statistical data on infectious diseases in a country, observing the incidence among travelers returning from the country can be considered as an alternative to estimate the status of infectious diseases in that country. Fukusumi et al. [9] showed that the incidence of dengue fever among Japanese travelers who returned from dengue-endemic countries was correlated with the incidence among the respective dengueendemic countries, mirroring the epidemic trends in those countries [9]. South Koreans are forbidden to travel freely in North Korea. However, from 1998 to 2008, tours to Mount Kumgang in the north of the eastern DMZ were permitted. From 2005 to 2015, the Kaesong Industrial Complex, a joint industrial zone of North and South Korea located in the northwestern part of the DMZ, was operated, allowing South Koreans to reside in or visit this area (Figure 1). During that period, malaria cases were reported annually among South Korean tourists to Mount Kumgang and South Korean employees at the Kaesong Industrial Complex. The cases were confirmed by microscopic examinations and were reported through the national mandatory reporting system, guaranteeing high accuracy and reliability of data.

This study aimed to develop hypotheses on the trend of malaria incidence in provinces adjacent to the DMZ in North Korea using malaria incidence data among South Korean tourists to Mount Kumgang and South Korean employees at the Kaesong Industrial Complex. We also aimed to provide a small window into the status of malaria in North Korea.

## MATERIALS AND METHODS

### Data sources

The number of South Korean tourists visiting Mount Kumgang from 2000 to 2008 was obtained from an open national statistical portal, the Korean Statistical Information Service (KOSIS) [10,11]. In mid-July 2008, South Koreans stopped touring Mount Kumgang, so for 2008, only the data up to June were used. The yearly number of tourists in 2008 was estimated using 2006 and 2007 data, which were provided in the form of the monthly number of tourists. The number of South Korean visitors to the Kaesong Industrial Complex and the number of South Korean employees residing in the Kaesong Industrial Complex from 2005 to 2015 were also obtained from the KOSIS [12,13]. All data were stated to have been obtained from sources in the Ministry of Unification of South Korea. The number of malaria cases among South Korean tourists to Mount Kumgang and South Korean employees at the Kaesong Industrial Complex was obtained a previous report [14].

The number of malaria cases in South Hwanghae Province, North Hwanghae Province, and Kangwon Province in North Korea from 2004 to 2016 was obtained from a paper published by Kim et al. [5]. The number of North Korean population by province was obtained from a national report of North Korea (the 2008 population census) provided by the United Nations Population Fund [15]. The annual prevalence of malaria per 1,000 population in Saenal-ri (village), Hwangju-eup (town), from 2001 to 2003 was obtained from a paper published by Chol et al. [16].

### Calculation of malaria incidence

To compare trends in malaria incidence between South Korean data and North Korean data, the malaria incidence per 100,000 person-years was calculated. South Korean tourists to Mount Kumgang were assumed to stay at Mount Kumgang for at least 48 hours because most of the tour packages consisted of 2 nights and 3 days. The person-years of the tourists were calculated for each year according to the following formula: [person-years_Kumgang_= number of tourists× 2/365]. For South Korean employees at the Kaesong Industrial Complex, the number of employees commuting daily from South Korea to the Kaesong Industrial Complex was estimated by dividing the annual number of visitors to the Kaesong Industrial Complex by 365 because the annual number of visitors was counted with the allowance of duplicates. The commuters were assumed to stay at the Kaesong Industrial Complex for 8 hours a day (i.e., one-third of a day), and the employees residing in the Kaesong Industrial Complex were assumed to stay year-round. The person-years of employees at the Kaesong Industrial Complex were calculated for each year according to the following formula: [person-years_Kaesong_= number of commuters × 1/3+the number of resident employees]. Using the calculated person-years and the number of malaria cases, malaria incidence per 100,000 person-years was calculated for each year. For North Korea, the number of malaria cases per 100,000 population in each province was calculated for each year.

### Ethics statement

The study was approved by the Institutional Review Board (IRB) of Sungkyunkwan University (IRB No. SKKU 2018-01-005).

## RESULTS

Table 1 shows the estimated number of South Korean tourists to Mount Kumgang and the estimated number of South Korean employees in the Kaesong Industrial Complex from 2000 to 2015. Table 2 shows the incidence of malaria per 100,000 person-years among South Korean tourists to Mount Kumgang and South Korean employees in the Kaesong Industrial Complex. The incidence per 100,000 person-years among Mount Kumgang tourists increased three-fold from 514 in 2000 to 1,577 in 2001, and then declined dramatically to 215 in the following year. In 2006, the incidence was 1,479, similar to that in 2001. The incidence decreased to 529 in 2007. The incidence per 100,000 person-years among employees in the Kaesong Industrial Complex nearly doubled from 2,388 in 2005 to 4,134 in 2006. It then dropped to 586 in 2008 and consistently declined, reaching 107 in 2015. The incidence of malaria among South Korean employees in the Kaesong Industrial Complex and South Korean tourists to Mount Kumgang showed a positive correlation between 2005 and 2008 (r= 0.729).

**Figure 2.**
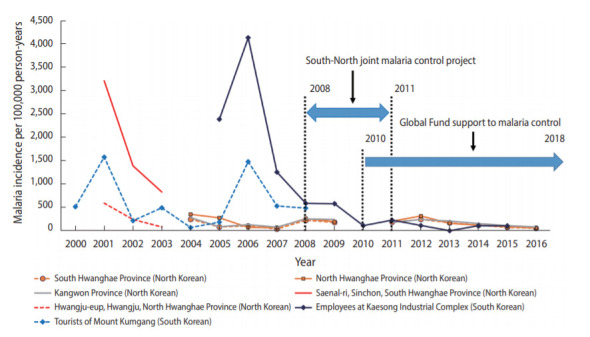
Malaria incidence in South Koreans visiting North Korea versus malaria incidence in North Koreans.

Table 3 shows the incidence of malaria per 100,000 population from 2001 to 2016 in South Hwanghae Province, North Hwanghae Province, Kangwon Province, Saenal-ri in South Hwanghae Province, and Hwangju-eup in North Hwanghae Province. The incidence of malaria in Saenal-ri in 2001 was 3,210 per 100,000 population and decreased rapidly in the following years to 830 in 2003. The incidence of malaria in Hwangju-eup in 2001 was 590 per 100,000 population and decreased to 80 in 2003. From 2004 to 2012, the incidence of malaria in the three provinces adjacent to the DMZ slightly increased or decreased, with an average of 188 per 100,000 population; it was higher than 200 in 2004, 2008, and 2012. The incidence of malaria gradually decreased after 2012. Figure 2 graphically shows the trends of malaria incidence among South Korean visitors to North Korea and North Koreans residing in provinces near the DMZ.

## DISCUSSION

Since North Korea reported a re-emergence of malaria in 1998, the incidence of malaria surged and peaked in 2001, with a total of 601,013 cases from 1999 to 2001 [3]. The resurgence of malaria is thought to be related to the North Korean economic crisis in the mid-1990s, which was aggravated by a series of natural disasters [17]. Malnutrition and reduced immunity of hosts, changes in the mosquito ecosystem caused by flooding, and lack of preparedness to combat malaria may have contributed to the spread of malaria. The lack of preparedness was manifested as a lack of diagnostic equipment, anti-malarial drugs, and trained personnel [3,17,18]. The WHO started the mass primaquine preventive treatment (MPPT) program in North Korea from 2002, and this markedly reduced the incidence of malaria. Both South and North Korean data showed this trend, especially in Saenal-ri in North Korea.

In 2006, the incidence of malaria among tourists to Mount Kumgang and employees of the Kaesong Industrial Complex increased sharply and was similar to that of 2001. Although the North Korean data did not show this increase, the following considerations support the possibility of a surge in 2006. From 2002 to 2006, the annual number of cases treated with MPPT in North Korea was about 400,000. However, in 2007, MPPT for about 5,000,000 people was requested and implemented [3]. This may suggest that there may have been a considerable increase in malaria cases in 2006, which may have been linked with floods and the negative North Korean economic growth rate in 2006 [19,20]. Worsening economic situations are known to have an impact on the spread of infectious diseases, such as the re-emergence of malaria following the economic crisis in Greece in 2009-2012 and the re-emergence of malaria in North Korea mentioned above [21]. Meanwhile, considering that the malaria trend in North Korea is related to that in South Korea, the increase of malaria cases in South Korea in 2006 supports the possibility of an increase of malaria cases in North Korea in 2006, as estimated from visitors’ data [2].

Based on the incidence of malaria among South Koreans who visited North Korea, the hypothesis that there were surges in malaria incidence in North Korea in 2001 and 2006 is plausible. North Korean data also showed a high incidence in 2001 and a rapid decline in the following years. However, the incidence of malaria in North Korea was relatively low and stable from 2004, which does not align with the trend among South Koreans who visited North Korea.

In 2008 and 2012, the incidence of malaria in North Korea increased slightly, which may have resulted from the cessation of the MPPT in 2008 and 2012 [3]. In addition, flooding that occurred in 2007 may have contributed to an increase of malaria in 2008 [22]. Overall, the data suggest that since 2009, the incidence of malaria has gradually decreased among both South Korean visitors to North Korea and North Koreans residing near the DMZ. This can be considered to reflect the effect of the South-North Korean joint malaria control project supported by Gyeonggi Province, Incheon, and Gangwon Province from 2008 to 2011, as well as the malaria eradication program of the Global Fund that began in 2010.

The limitations of this study are as follows. First, the malaria incidence among South Koreans who visited North Korea was calculated using the estimated person-years under several assumptions. However, this was probably not a significant problem because this study was designed to examine trends in incidence, rather than the exact incidence. Second, other factors that could affect the malaria incidence among visitors, such as whether they lived in high-risk areas of malaria in South Korea and changes in the rate of taking preventive antimalarial medicine, were not considered in the study due to a lack of Dinformation on the visitors. Third, according to the study that provided the incidence for each province in North Korea [5], data from 2004 to 2009 were obtained through international conference reports, press releases of the Korea Centers for Disease Control & Prevention, and newspaper articles because the number of malaria cases by region in North Korea was not officially disclosed. Given the characteristics of the North Korean regime, access to North Korean data is difficult, and therefore, it is challenging to evaluate the validity of data from North Korea [23,24].

The strength of this study is its uniqueness. This study examined trends in malaria incidence in North Korea using South Korean visitors’ data and simple calculations. The study is meaningful because it used a proxy indicator for malaria monitoring in North Korea. This approach to monitoring can be used when the relationship between South Korea and North Korea improves and visitor exchanges resume.

In conclusion, the incidence of malaria in North Korea increased explosively in the early 2000s but has since decreased through international aid. In order to maintain a consistently low malaria incidence, the South-North Korean joint malaria control project and support of the Global Fund should continue [25,26]. We propose the establishment of a North Korean malaria monitoring system using the incidence among South Korean visitors to North Korea for future inter-Korean collaboration aiming to eradicate malaria.

## Figures and Tables

**Table 1.** Number of South Korean visitors to North Korea from 2000 to 2015

Year	Mount Kumgang tourists	Kaesong Industrial Complex	
		Commuters^1^	Resident employees
2000	213,009	-	-
2001	57,879	-	-
2002	84,727	-	-
2003	74,334	-	-
2004	268,420	-	-
2005	298,247	112	507
2006	234,446	167	791
2007	345,006	274	785
2008	452,686^2^	418	1,055
2009	-	306	935
2010	-	337	804
2011	-	314	776
2012	-	329	786
2013	-	208	757
2014	-	345	815
2015	-	352	820

1 South Korean employees who commuted daily from South Korea to the Kaesong Industrial Complex.

2 The number of tourists in 2008 was estimated using 2006 and 2007 data.

**Table 2.** Malaria incidence among South Koreans visiting North Korea from 2000 to 2015

Year	No. of malaria cases		Person-years		Malaria incidence^1^	
	Mount Kumgang	Kaesong Industrial Complex	Mount Kumgang^2^	Kaesong Industrial Complex^3^	Mount Kumgang	Kaesong Industrial Complex
2000	6	-	1,167	-	514	
2001	5	-	317	-	1,577	
2002	1	-	464	-	215	
2003	2	-	407	-	491	
2004	1	-	1,471	-	68	
2005	3	13	1,634	544	184	2,388
2006	19	35	1,285	847	1,479	4,134
2007	10	11	1,890	876	529	1,255
2008	12	7	2,480^5^	1,194	484	586
2009	5^4^	6	-	1,037		579
2010	-	1	-	916		109
2011	-	2	-	881		227
2012	-	1	-	896		112
2013	-	0	-	826		0
2014	-	1	-	930		108
2015	-	1	-	937		107

1 Malaria incidence per 100,000 person-years.

2 Person-years=number of tourists×2/365 (48 hr/365 d).

3 Person-years=number of commuters×1/3 (8 hr/24 hr)+number of resident employees.

4 Cases with a long incubation period.

5 The number of tourists in 2008 was estimated using 2006 and 2007 data.

**Table 3.** Malaria incidence in provinces near the Demilitarized Zone in North Korea from 2001 to 2016^1^

Year	South Hwanghae	North Hwanghae	Kangwon	Saenal-ri in South Hwanghae	Hwangju-eup in North Hwanghae
2001	-	-	-	3,210	590
2002	-	-	-	1,390	240
2003	-	-	-	830	80
2004	243	351	281	-	-
2005	82	276	84	-	-
2006	100	71	130	-	-
2007	35	69	77	-	-
2008	222	260	252	-	-
2009	178	193	241	-	-
2010	-	-	-	-	-
2011	185	205	179	-	-
2012	242	315	240	-	-
2013	169	159	207	-	-
2014	117	116	152	-	-
2015	72	89	107	-	-
2016	52	59	81	-	-

1 Malaria incidence per 100,000 population.
